# Clinical Verifications of Pyrogenum

**Published:** 1893-04

**Authors:** G. W. Sherbino

**Affiliations:** Abilene, Texas


### CLINICAL VERIFICATIONS OF PYROGENUM.

G. W. SHERBINO, M. D., ABILENE, TEXAS.

In the two years that I have had since the proving of Pyrogenum I have collected some of the symptoms together, so that my colleagues could receive the benefit of this experience.

The hard bed and the hard pillow and the intense aching that they sometimes compare to lying on a pile of rocks, shows the extreme soreness of Pyrogenum; sometimes the patient declares that a train of cars has run over him. Pyrogen has *fan-like motion of the alæ nasi*, Antimonium-tart., Baptisia, Bell., Bromium, Hell., Lyc., Phos., Rhus-tox. all have it. I can remember when I knew of no others but Lycopodium, but it is well to have them in mind, as very often it is a great help to know these things. Great restlessness is better when first commencing to move (Rhus-tox., worse when commencing to move). This is the difference between Rhus and Pyrogenum. It took me some time to find it out, as the restlessness was so much like that of Rhus-tox. that it took very close observation to detect the real difference. It has that death-like restlessness or the restlessness as of death or those in *articulo mortis*, amelioration from sitting up in a chair and rocking hard; the amelioration from moving lasts only a few moments, and this necessitates that the patient keep on moving, as the amelioration is but momentary.

Throbbing of the vessels of the neck. The carotids have a distinct wave-like throb, similar to Bell. and Spigelia. The throbbing is from the clavicles upward. There is numbness of the hands and arms; numbness of the feet, and the numbness extends over the whole body.

Violent heart action, keeping up so that it was very tiresome; delirious when closing the eyes; sees a man at the foot of the bed or in the farther part of the room; hands cold and clammy; bowels so sore that she can hardly breathe; bowels so sore cannot bear any pressure over the right side; aggravation from sitting up in bed; dizziness on rising up in bed like Baptisia, Bryonia, and Phytolacca (Rhus-tox.).

Tongue coated white; yellow-brown streak down the centre like Baptisia.

Symptoms of paresis: The child could not walk; was sitting on the edge of the bed and waving her body back and forth—was relieved when in motion.

Vomiting water when it becomes warm in the stomach like Phosphorus—sick stomach better by drinking very hot water.

Vomiting ameliorates the sick stomach. Antimon-tart., Ipec. go to sleep after vomiting. Coughing up rusty colored mucus; pain in the right lung and shoulder, aggravated from coughing or talking; circumscribed redness of both cheeks; palpitation of the heart, with abnormal temperature, pulse 160 per minute.

Palpitation worse from motion, better from remaining quiet. Inclined to talk all the time at night during the fever; cold sweat over the body; pain in the small of the back; desires to urinate; it is scant; talks to herself; tries to vomit; urging to vomit, with cold feet; restlessness relieved after sleeping; purple spots on the chest; cries out in her sleep that some one or a weight is lying on her; heart beats hard, has a laborious action; sensation as if the heart was too full of blood; it beats very loud, heart sounds can be heard a foot away from the thorax (always can hear her heart beat); could not sleep last night for heart whizzing and purring so, when she did drop off to sleep she was delirious; sensation as if a cap were on her head. When she awakens and finds this cap on her head she knows that she is all right, that she is not delirious; better after vomiting; whispers in her sleep; whispers to herself, if you ask her what she said she does not answer; sensation as if she covered the whole bed; she knew that her head was on the pillow, but she could tell where the rest of her body was.

She feels when lying on one side that she is one person and that when she turns to the other side she is another person; sensation as though the fever would not run in each alike, that is to say, she felt as though she was existing in a second person, or that there were two of her. This is like Baptisia, the fever wants to run separate.

Tongue dry and not a particle of moisture on it. Has had no thirst since she has been sick. Bitter taste in the mouth; tongue dry down the centre.

Coldness and chilliness all day that no fire would warm; sits by the fire and breathes the heat from the stove; chilly whenever leaving the fire; at night when the fever came on he had a sensation as if his lungs were on fire and that he must have fresh air, which soon brought relief.

Sensation as though he was crowded with arms and legs; when turning over in bed they were still crowding him; as soon as the fever came on he commenced to urinate; he can tell every time when the fever is coming on because of this frequency to urinate. The urine is as clear as spring-water. Very severe pain in the right side; knife-like pains going through to the back; worse from every motion, from coughing or talking or taking a long breath; better from lying on the affected side (Bryonia), Baptisia; groaning with every breath; redness of the face, and also of the ears, it looked as if the blood would burst out of them. After the fever leaves he still has the hallucination that he is very wealthy and he has a very large sum of money in the bank, and this was the last to leave him—this idea that he had the money.

Child with cerebro-spinal meningitis was so sick that at one time it seemed as though she could not recover; there was automatic motion of the right arm and right leg; this kept up until it would turn her around from left to right till her feet would get on the pillow or touch the head-board; she was brought out of this conditiou with Pyrogenum.

Rolling the head from side to side. It has cured prolapsus uteri, with bearing down, relieved by holding the breath and straining, as in the act of labor. Pain starting in the uterine region and passing upward to the umbilicus (Sepia). It was the reverse (a cork-screw pain) this was momentarily relieved by holding the breath and bearing down, as in labor; momentarily relieved by pressing the hands against the vulva, then she said she would have to turn loose, as it made her worse. Lillium had previously failed in this case. Pain starting in the umbilicus or a little above, and passing down toward the uterus, but at midway of the distance to the uterus it would be intercepted by the same kind of a pain starting from the uterus and passing upward till they would meet midway between the umbilicus and uterus, then gradually die out till another would come as before; ameliorated momentarily by drawing her knees up to her chin and grasping her arms around them and holding them tight. Intolerable tenesmus of the bladder, it was more of a spasmodic contraction than anything else; at the same time, this condition would be reflected to the rectum and this strong desire to defecate without ability to do so would also involve the ovaries and broad ligaments, Nux-vom. and Lilium-tig. had no affect whatsoever. This was a Morphine case from beginning to end.

I have now had two years' experience with this grand nosode, and it is one of the grandest monuments to Hahnemann and to Homœopathy, as it covers a wide range of action, and it fills a place of its own that no other remedy can fill.

### PYROGENUM$^{CMM}$.

I was called on the 2d day of January, at one o'clock P. M., to see girl æt. fourteen, who had been suffering for about one week, and for the last two days had been attended by an allopath. I soon diagnosed the case, for the benefit of the family and the neighbors, to be that of pneumonia. I found the temperature 105½° ; respirations, 52 per minute; pulse, 120 ; coughing up rusty-colored mucus ; pain in the right lung and shoulder, worse from coughing or talking; circumscribed redness of both cheeks ; *fan-like motion of the alœ nasi.* (Ant-t., Bapt., Brom., Bell., Lyc., Phos., Rhus, Pyrogenum.) Restlessness, when first beginning to move, restlessness all night.

January 2d.—Temperature, 102½° ; pulse, 104 ; much better every way ; improving fast.

January 3d.—Temperature, 100° ; pulse, 96 ; respiration, 32.

January 4th.—Temperature, 97½° ; pulse, 80. Discharged the case cured. She took four doses of Pyrogenum$^{cmm}$.

### PYROGEN.$^{DMM}$.

Mrs. —— sent to the office for medicine for an aching all over; says she was so very restless last night, could not keep still. I sent her one dose of Pyrogen., which cured her by morning.

I was sent for to go and see a little girl that had been taken sick at night with symptoms of paralysis. She was well up to that time. When I arrived she could not stand on her feet; when they set her up in bed she would wave back and forward, as if she had no control of herself; her pulse was 120 per minute, her temperature was 98.3°. Taking the increased heart action as a leading symptom, I gave her a dose of the CMM of Pyrogen., which cured her quickly.

### PYROGEN. IN FEVER.

Mrs. A. was taken with a fever yesterday, and she had a miserable night last night; her temperature is 103°, and pulse 130; was very restless, especially after midnight, constantly changing her position in bed. ℞. Rhus-t.$^{cm}$. Called the next morning and found her no better, and had put in a bad night again, never sleeping at all; says that it keeps her busy trying to get into an easy position, and she noticed that she was better *while she kept up this motion all the time.* ℞. Pyrogen.$^{cmm}$. She commenced to improve at once, rested better at night, and all of the aching left, the pulse was 108 the last time I saw her, and at that time it was at normal temperature; she had a great deal of throbbing of the vessels of the upper thorax and neck. It would shake the bed it was so violent.

### CHILD WITH LA GRIPPE.

Her father called at the office for medicine, and said that she was very restless; worse from lying down, better sitting up.

Vomiting water after drinking as soon as it gets warm in the stomach (Phos.). Better by vomiting. Cured with one dose of Pyrogenum$^{cmm}$.

### La Grippe.

Mr. —— complains of a feeling of coldness and chilliness that no fire can warm. This kept up all day and all night. As night came on he grew more restless, and as he had been breathing hot air all day from the fires he now had an intense desire for fresh air, and thought if he did not get it his lungs would burn up. His respiration was quite quick, but the fresh air soon brought relief in that respect.

He groaned all night, and rolled and tumbled from one side of the bed to the other, not lying in one spot but a moment at a time. The bed was as hard as a board (Bapt.), the pillow was as hard, and he was as sore as if a train had run over him. He tried to lie on his face but he found that side of his body was sore too.

Before daylight he awoke and told his wife he was so glad that he had got rid of those arms and legs that had crowded him all night; if he turned over they were there, and he was trying all night to get them out of the bed. As soon as the fever began to come on he commenced to urinate, and he must have passed water every half-hour. The urine was as pale as spring water; the fever went down the next day; but when it commenced to rise again he commenced to have frequent urinations as before, and he knew that the fever was coming up as he was urinating so frequently. He took two or three doses of Pyrogenum$^{cmm}$.

### Dysmenorrhœa.

Mrs. W. F. F—— has had painful menstruation for several years, and it is always preceded by aching in the bones, causing her to complain of the bed being hard and accompanied by intolerable restlessness.

She is better when first beginning to move—and she has to keep this up, as it affords some *relief to* the restlessness. I saw her have one of these spells, and she was on the floor, and she would curl up and then straighten out, and then turn and twist in every possible position. The CMM of Pyrogenum would always relieve her, till now she does not have the aching, and is doing nicely. This remedy has cured this condition, but other remedies had to be used—Lac-can., Æscul-hip., and Helon.

### PYROGENUM$^{CMM}$.

I was called to see a colored girl about twelve years old, who seemed to be partially paralyzed. She could not sit up or stand nor walk a step without help. She was very restless and kept rocking back and forth while sitting on the edge of the bed. She said that this rocking motion relieved her and so she kept it up.

I gave her a dose of Pyrogenum$^{cmm}$ and she was all right in a day or two, as I only saw her but once.

### PYROGENUM$^{DMM}$.

Mr. M. H.—Child had had a fever for several days and was getting worse (Bell. and Rhus-tox. had failed). I sat up all night with the case and it had the restlessness of death. There was more motion of the right leg and arm, and she would make a semicircle from left to right, and her feet would get upon the pillow and she was not still for one moment all night long. She would make this peculiar circle and would have to be put on the pillow, and in a very short time she would be kicking the headboard with her feet. All of these symptoms passed away under the action of Pyrogenum$^{dmm}$.

Mrs. M. has been troubled for some time with bearing-down feelings in the uterine region, relieved by holding her breath and pressing down as in labor. She was very restless at night and had to keep in motion, as only then could she get any relief (better when first beginning to move).

Cured by Pyrogenum$^{dmm}$.

### PNEUMONIA, PYROGENUM$^{CMM}$.

January 4th, 1892.—I was called to see Mr. K., who took a severe chill yesterday and was chilly all night long. I found him expectorating rusty-colored blood this morning. He complains of aching all over and his eyeballs are sore and his ears are sore. The bed is hard and he aches in all the bones in his body. He groans all night and he was restless. He is worse from moving at first, but better after he gets quiet again, and then after lying long in one place he is compelled to move again. Rhus-tox.$^{cm}$, one dose. Temperature, 102½° ; pulse, 100 ; respiration, 32.

I called in the evening at five o'clock. Temperature, 103.3°.

January 5th.—He had a bad night, his wife could hardly keep him in the bed ; he was groaning and restless. He had to keep moving all the time to get a little ease. He felt as if a train had run over him and bruised him badly. He took two doses of Pyrogenum$^{cmm}$ in the night.

January 5th.—I find him better this morning. Temperature, 100° ; pulse, 80 ; respiration, 24.

January 6th.—I found my patient had eaten some hot tomatoes and at one o'clock A. M. he commenced vomiting and is now having cramp in the stomach. Gave a dose of Nux$^{cm}$, as it covered his symptoms well. Temperature and pulse going up again.

January 7th.—He had a very restless night of it last night. He ate some fresh oysters yesterday and they disagreed with him and kept his stomach in an agitated condition. He complains of feeling so full after eating, and has fan-like motion of the alæ nasi and he thinks he is worse in the after part of the afternoon. I gave him Lyc.$^{mm}$ (H. S.), one dose, dry.

January 8th.—He was very restless last night. Temperature, 103° ; respiration, 32 ; pulse, 100. I called at night and he was sitting up and he had a restless spell about half an hour before I called and was still in the same condition. He could not sit still or keep in one position a half-second without changing ; this he said was all the relief he could get. He was better while moving, but the relief lasted only a moment and he had to keep changing in that way continually. I gave him a dose of Pyrogenum$^{cmm}$ dry on the tongue. This was all the medicine required, and I discharged him on the 11th cured.

November 29th.—I was called to see Mrs. R., æt. twenty-one, who was taken sick last night while at the opera, and had to leave as she felt as if she was going to faint. She left with her husband, and she says that she knew nothing while going down the stairs as it was all a blank to her. As soon as she reached the carriage she fainted, and did not come to her senses till she got near home; at about five o'clock she was taken with a hard chill and shook for about an hour, with chattering of the teeth. She complains of aching all over; the bed feels hard, also the pillows (Bapt., Rhus-t.) fan-like motion of the alæ nasi (Ant-t., Bapt., Brom., Lyc., Phos.) Temperature, 105°; pulse, 126; respiration, 44. The heart action was so strong that her clothing had a distinct vibration on the neck and thorax; throbbing of the arteries of the neck most noticed in the lower part of the clavicles, upwards. Numbness of the hands and arms (Bapt., Rhus). ℞ Baptisia$^{600}$ (M. S. G.).

November 30th.—Called this morning and found that she had had a bad night, was restless, worse after midnight. She could not lie but one moment in one place in the bed, there was momentary amelioration from motion; as soon as she would stop moving she got so restless she could not stand it, and had to keep this up all the latter part of the night. She complained that the heart action kept up, throbbing so violently that she got very tired of it; aching worse than yesterday; is delirious this morning when she closes her eyes. Temperature, 105°; pulse, 126; respiration, 44; this was at 10 A. M. Taking the rapidity of the heart's action; the amelioration from constantly changing the position; amelioration during act of moving (Rhus-tox., aggravation when first beginning to move), I gave Pyrogen.$^{omm}$ (Swan), one dose at 10 A. M. I took the temperature at 11 A. M., and it had come down to 104°; at 4 P. M. the temperature was 103.3°; was not aching so hard, not so delirious.

December 1st.—Called this morning at 9 A. M., found the temperature, 99.4°; pulse, 80; respiration, 28; says she has had a bad night; was very restless as she was night before, was unable to tell where she was, and that she made her husband get up several times to see if a man was in the house, she could see a man standing by the bed or at the farther part of the room; hands cold and clammy. Bowels acted several times last night. Her bowels are so sore that she can't bear the least pressure over the right side. She can't sit up they pain her so; she gets dizzy whenever she rises up in the bed (Bap., Bry., Phyt.). Tongue coated white, has had nothing to eat during this sickness, but has wanted nothing but water.

December 2d.—I left a dose of the Pyrogen. to take last night if she did not rest better, and it made her rest better. She feels pretty well this morning; has no appetite yet. Tongue not coated so much. Temperature, 98.3°; pulse, 64; respiration, 24.

December 3d.—Is not quite so well this morning as she feels a little aching, has no appetite; temperature, 100°; pulse, 68.

December 4th.—Called and found her sitting up and has no fever and I discharged her cured.

### Typhoid Pneumonia.

January 22d.—Was called in about three A. M. to see a man who had taken a chill in the night and very severe pain in his right side, the pain starting in the lower part of the chest and going through to his back, worse from every motion; from coughing or taking a long breath; relieved from lying on the affected side (Bap., Bry.); this pain was knife-like and was very severe; he groaned with every breath. ℞. Bryonia$^{5cm}$.

Called at ten A. M., found the pain all gone, pulse, 100; temperature, 102°; respiration, 30. He is very stupid and drowsy, and will not keep awake long enough to get his symptoms. Aches all over. ℞. Bapt.$^{600m}$ (S. G.), one dose.

January 23d.—Was called at ten P. M. as he was so restless; he could not keep still. The pain had come back again in his side. I sat myself down to watch him and he was in constant motion. He was relieved when first beginning to move. Coughing and groaning; temperature, 102°; pulse, 100; respiration, 30. *Fan-like motion of the alœ nasi;* aching worse; has been delirious all day; does not know that I have been to see him; tongue covered with a very thick white coat; it is as white as milk; fetid breath. ℞. Pyrogen.$^{cmm}$ (Swan).

24th.—Very much worse; had a very miserable night; did not rest any. Temperature, 104°, at six A. M. Respiration, 30; pulse, 100.

Incoherent talking in his sleep; terrible throbbing of the carotid arteries from the sternum up to the head and neck; throbbing of the temporal arteries; face very flushed, and the ears were very red, as if the blood would burst out of them. The intense restlessness kept up all night, and in fact it was worse after taking the medicine. His bowels had moved, and they had him up before I arrived, and the mother thought it was the bowels moving that relieved him, but I did not think so. I thought it was the getting out of bed that gave him the relief. He became more quiet and had a fair day. He is still very stupid; he answers questions, but goes immediately back into his stupor again; has been snoring all day whilst asleep.

January 25th.—He had not so good a night last night; gave him a dose of the DMM (Swan). He is not so stupid to-day; did not know that I had been attending to him or that I had been about the house. Pulse, 92; temperature, 103.3°; respiration, 30. Coughing almost incessantly all day long; relieved immediately by getting in a chair; aggravation as soon as he lies down again; does not ache so much when sitting; is conscious to-day and knows what he is talking about.

January 26th.—Very much better to-day; the temperature, 100°; pulse, 80; coughs up a stringy mucus that looks as if mixed with iron-rust. He has been expectorating this all day.

January 27th.—Found him with no fever this morning, but coughing up large quantities of rusty-colored mucus. He seems all right, but upon one point he is off—that is, no sort of persuasion can make him think but that he has lots of money in the bank; talks about his big pile of money.

January 28th.—He slept well last night, not coughing so much this morning; tongue is cleaning off, and he says he feels pretty well to-day; is sitting up. I discharged him cured. He still thinks he has the money in the bank.

Mrs. —— was confined under the care of one of the materialistic school. She, at the time, having a very severe cough, her husband called at the office to get a remedy for after-pains. I was called in the morning and found the patient in the following condition: She had had a very restless night, coughing up rusty-colored sputa; rapid fan-like motion of the alæ nasi; respirations, 84 per minute; pulse, 140; temperature, 102°; called in the evening at four P. M.; respirations, 100 per minute; pulse 140; temperature, 102°; she was very restless and found relief while she was moving; pain in each hypochondrium when coughing; called at nine P. M.; pulse, 144; respirations, 96; temperature, 101°.

April 3d.—Respirations, 72; pulse, 132; temperature, 99.3°; called at eight P. M.; respirations, 54; pulse, 124; temperature, 99°.

April 4th.—Temperature, 98.2°; pulse, 116; respirations, 34; she feels well this morning; rested well last night. I am now sure she will recover. She has taken three doses of Pyrogenum$^{cmm}$ in a glass half full of water, and taken at once S. L. every half-hour while awake.

### Relapse from Typhoid Fever.

Mrs. L., aet. sixty, had just gotten over a spell of fever when one morning she was taken with a palpitation of the heart; her pulse was running at the rate of 160 per minute, and the temperature about normal. I thought from the condition it called for Digitalis, as she was so afraid to move, but it did her no good; the next night she began to ache all over in the bones, and was very restless. I tried Bapt. and then Rhus-tox. without any benefit whatever. The fever kept climbing up each day till it got to 104.3°, and this was always in the after part of the night, about two or three o'clock A. M. *She always felt best when the fever was on*, but as soon as the fever left the aching came again. Bapt. and Rhus-tox. have aching worse when the fever is on. There was one peculiar thing about the temperature. Her daughter took her temperature about every two hours through the twenty-four, and it would vary from one to two degrees each time; this was so during the inclination and the declination.

October 14th.—Had a very restless night last night. She says she could not lie still but a few seconds, as she must keep moving all the time, and felt better from doing so. At nine A. M. tried to vomit; has no appetite nor any thirst; feels deathly sick at the stomach. Gave one dose of Pyrogen.$^{cmm}$ (Swan).

October 15th.—Pulse, 120; temperature, 104.3°; bed still feels hard; aching all over; still restless, worse after midnight, till morning; better from constantly changing the position.

October 17th.—Rested better last night after taking a dose of the CMM; inclined to talk all the time in an excited way; cold sweat over the body.

October 19th.—Pulse, 88; temperature, 104; pain severe in the small of the back; desires to urinate; it is scant; talks to herself; tries to vomit.

October 21st.—Pulse, 84; temperature, 103°; strains to vomit; cold feet. Restlessness is better as she sleeps some.

October 23d.—Getting better. Purple spots on the chest; pulse, 80; temperature, 103°; cries out in her sleep that a heavy weight is resting upon her; more restless last night. Gave a dose of Pyrogen.$^{cmm}$.

October 26th.—Pulse, 72; temperature, 101°; heart beats hard, has a laborious action; sensation as if the heart were too full of blood; it beats very loud; heart sounds can be heard a foot away from the thorax (always can hear her heart beat); fan-like motion of the alæ nasi (Ant-tart., Baptis., Bromin., Bell., Lyc., Phos., Pyrog., Rhus-tox.).

October 28th.—She could not sleep last night for the heart whizzing and purring so; when she did drop off to sleep she was delirious; sensation as if a cap were on her head; when she awakens and finds this cap on her head she knows that she is all right—that is, she is not delirious; better after vomiting; whispers in her sleep; whispers to herself; if you ask what she has said she does not answer; sensation as if she covered the whole bed; she knew that her head was on the pillow, but she could not tell where the rest of her body was. She feels when lying on left side that she is one person, and when she turns to the other side that she is another person; she can't get the fever to run in *the other person* like herself; the fever wants to run separately; tongue dry, is not a particle of moisture on it.

October 31st.—Bitter taste in the mouth; tongue dry down the centre; has had no thirst since she has been sick. Pulse, 76; temperature, 98.5°. Discharged her cured.

